# Attention and Working Memory Biases to Black and Asian Faces During Intergroup Contexts

**DOI:** 10.3389/fpsyg.2018.02743

**Published:** 2019-01-09

**Authors:** Guadalupe D. S. Gonzalez, David M. Schnyer

**Affiliations:** Cognitive Neuroscience Laboratory, Department of Psychology, The University of Texas at Austin, Austin, TX, United States

**Keywords:** working memory, racial bias, ERP, own-race bias, intergroup contexts

## Abstract

Categorizing and individual as a racial ingroup or outgroup member results in processing and memory differences. However, despite processing differences for racial ingroups and outgroups, very little is known about processing of racial ingroup and outgroup members during intergroup contexts. Thus, the present research investigated attention and memory differences for racial ingroup and outgroup members during competition for attention (i.e., intergroup contexts). In experiment 1, event-related potentials (ERPs) were obtained while participants completed a working memory task that presented 4 faces (2 Black, 2 White) at once then, following a short delay, were probed to indicate the spatial location of one of the faces. Participants showed better location memory for Black than White faces. During encoding, ERP results revealed differences based on the race of the face in P300 amplitudes, such that there was greater motivated processing when attending to Black faces. At probe, the N170 indicated enhanced early processing of Black faces and greater LPCs were associated with better recollection of Black face location. In a follow-up study using the same task, we examined attention and working memory biases for Asian and White faces in Caucasian and Asian participants. Results for both Caucasian and Asian participants indicated better working memory for Asian relative to White faces. Together, results indicate that during intergroup contexts, racial minority faces capture attention, resulting in better memory for those faces. The study underscores that examining racial biases with single stimuli paradigms obscures important aspects of attention and memory biases during intergroup contexts.

## Introduction

Individuals tend to process racial ingroup and outgroup members differently and these differences have important implications for intergroup relations (Marcon et al., 2010; DeGutis et al., 2013; Hayward et al., 2013; Kawakami et al., 2014, 2018). For example, research shows that individuals tend to remember faces of their own race better than those of other races (i.e., own-race bias, cross-race effect) (for a review see Meissner and Brigham, 2001; Walker and Tanaka, 2003; Walker and Hewstone, 2006; Mondloch et al., 2010; Zhao et al., 2014; Wan et al., 2015). While a large body of research has examined memory biases for racial ingroups and outgroups, fewer research has focused on attention biases to these groups. Examining attention biases to racial ingroups and outgroups is important because it has been implicated as a contributing factor in phenomena such as the own-race bias and interaction intentions with outgroup members (Hills and Lewis, 2006; Hugenberg et al., 2010; Hills and Pake, 2013; Kawakami et al., 2014, 2018; Zhou et al., 2015a,b). Additionally, some research suggests that early attention biases to Black relative to White faces may reflect an early vigilance to Black individuals due to stereotypes associating Black people with danger or threat (Trawalter et al., 2008). Despite the important role of attention biases to racial ingroups and outgroups, little is known about how individuals deploy attention when multiple races are present. Contexts in which faces from multiple races are present are more likely to illuminate the way in which race interacts with cognitive processes during intergroup contexts. Therefore, the current research sought to examine whether existing effects associated with own-race biases in attention and memory persist in contexts where faces from multiple races are simultaneously present.

A variety of factors have been proposed to explain processing differences between racial ingroup and outgroup members including perceptual expertise, social categorization, and motivation (Rhodes et al., 1989; Valentine, 1991; Tanaka et al., 2004; Meissner et al., 2005; Walker and Hewstone, 2006; Bernstein et al., 2007; Rhodes et al., 2009; Hugenberg et al., 2010; for a review see Young et al., 2012). For instance, socio-cognitive theories propose that categorization of individuals as members of an ingroup or outgroup results in processing differences and reduced memory for outgroup members (Levin, 2000; Shriver et al., 2008; Hehman et al., 2010; Hugenberg et al., 2010; Shriver and Hugenberg, 2010). In particular, the race-feature hypothesis (Levin, 1996, 2000) proposes that racial outgroup faces are encoded on the basis of race-specifying features, whereas racial ingroup faces are encoded on the basis of individuating features. Thus, attending to race-specifying features of racial outgroup faces is an optimal search strategy because it speeds up detection of racial outgroup members. On the other hand, greater individuation of racial ingroups would result in better memory for racial ingroup members. Altogether, the race-feature hypothesis predicts differences in memory and visual search for ingroup and outgroup members.

Research on the race-feature hypothesis has examined individuals’ ability to search for and detect racial ingroup and outgroup faces using visual search tasks (Levin, 1996, 2000; Chiao et al., 2006; Lipp et al., 2009; Sun et al., 2013). In visual search tasks individuals are instructed to indicate the presence or absence of a target face (own- or cross-race) among an array of distractor faces of the opposite race; thereby, incorporating competition for attention between races. However, research has yielded mixed support for a cross-race search asymmetry (Levin, 2000; Sun et al., 2013; Lipp et al., 2009). For example, while Levin (1996) found that White participants were faster at detecting Black faces among an array of White faces, the same effect was not found for African American participants. On the other hand, Chiao et al. (2006) investigated whether a cross-race search asymmetry existed in Black, White, and Black-White biracial individuals. Results indicated that all participants, regardless of race, were faster and more accurate at detecting Black faces among an array of White faces than vice versa, suggesting that during competition between races, Black faces tend to capture attention. It has been proposed that the inconsistent findings on the cross-race search asymmetry could be due to differences in the stimuli used, as well as, cultural differences in processing faces (Lipp et al., 2009; Sun et al., 2013). Nevertheless, research on visual search asymmetries for racial ingroups and outgroups highlights the importance of examining the effects of competition for attention between races on processing of racial ingroup and outgroup faces.

Multiple methodologies, such as event-related potentials (ERPs), have been deployed to further examine the perceptual mechanisms underlying processing differences between racial ingroups and outgroups (Stahl et al., 2008; Brebner et al., 2011; Herzmann et al., 2011; Sessa et al., 2012; Amodio et al., 2014; Cassidy et al., 2014; Wiese et al., 2014). The ERP methodology is advantageous because with good temporal resolution it can be revealed whether race influences earlier, more automatic perceptual processes, or the later, more deliberative processes. In general, research examining processing of racial faces reveal race effects across multiple ERP components such as the N100, N170, N200, N250, and P300 (Ito et al., 2004; Ito and Urland, 2005; Dickter and Bartholow, 2007; Kubota and Ito, 2007; Lucas et al., 2011). For instance, Brebner et al. (2011) used ERPs to determine how encoding categorical (e.g., skin color) versus structural information during face processing affected memory in Caucasian participants. In the study, the skin color and facial structure of faces were modified such that each face had a Black or White skin color and a Black or White facial structure. Participants revealed an own-race bias through greater memory for faces with White than Black skin color, but no accuracy differences by facial structure. Additionally, there were more negative N170s and N250s for faces with Black than White skin color, indicating that skin color effects seem to be the primary driver of early perceptual processing. Greater N170 amplitudes were thought to indicate the influence of greater feature-based processing for faces with Black skin color. Moreover, participants showed greater N200s to faces with White than Black skin color, indicating increased attention and processing for racial ingroup faces. Overall, the authors concluded that early categorization on the basis of skin color increases motivation to attend to ingroup faces, resulting in greater memory for own-race faces.

Other ERP research also indicates that race can quickly capture attention (Ito and Urland, 2003; Dickter and Bartholow, 2007; Ito and Bartholow, 2009). One particular study examined whether individuals would attend to race even when it was irrelevant to task goals by obtaining ERPs during a categorization task that presented competing racial stimuli as flankers (Dickter and Bartholow, 2007). Results for White participants showed an outgroup processing bias; larger P200s and P300s to cross-race than own-race faces revealed increased attention toward the racial outgroup. Conversely, larger N200s to own-race relative to cross-race faces suggested enhanced early processing of ingroup members. Hence, the ERP differences revealed an early race effect in which individuals quickly categorize a person as an ingroup or outgroup member based on race. Importantly, these findings indicated that competing racial stimuli can affect processing of ingroup and outgroup members even when race is irrelevant to task goals. While some research finds relatively automatic effects of race on attention, other research indicates that motivational changes can also influence attention to race (Brosch and Van Bavel, 2012; Correll et al., 2014). Particularly, research indicates that attention to race is flexible and may respond to top-down goals such that attention may be directed to one race (e.g., White) over another race (e.g., Black) depending on the perceiver’s goals (Correll et al., 2014). Thus, creating an intergroup context during the presentation of competing racial stimuli may result in different motivational goals that influence early attention to racial ingroup and outgroup faces.

Despite the role of attention in processing of own- and cross-race faces (Zhou et al., 2015a,b), few studies have examined how competition for attention between races during encoding (i.e., intergroup context) may influence memory. Much of the research on the own-race bias in memory uses “old/new” or “remember/know” paradigms that require individuals to encode one face at a time (Rhodes et al., 2009; Stahl et al., 2010; Brebner et al., 2011; Pezdek et al., 2012), which does not allow for the examination of how competition for attention affects recognition of own- and cross-race faces. Although some studies incorporate competition for attention during encoding to assess working memory for own- and cross-race faces, the paradigms used did not incorporate competition between different races (Sessa et al., 2012; Sessa and Dalmaso, 2016). For example, Sessa et al. (2012), obtained ERPs while participants completed a visual working memory task that cued them to memorize one or two faces (encoding presentations that were all-Black or all-White) presented in one visual hemifield. After a short delay, participants were presented with the same number of faces and asked to indicate whether the face(s) had changed. Surprisingly, behavioral results did not show significant differences in memory for White and Black faces. However, amplitude differences in the sustained posterior contralateral negativity (SPCN) between Black and White faces were greater for individuals who showed greater implicit prejudice on the Implicit Association Test, indicating that individuals with higher prejudice encoded Black faces with lower precision than White faces. A follow-up study indicated that eye-gaze direction may also influence working memory maintenance for Black and White faces (Sessa and Dalmaso, 2016). However, given that both studies always presented the same race at encoding, they did not examine what would happen when there is competition for attention between races.

While competition for attention between races has been incorporated in visual search tasks, the inconsistent findings regarding detection of own- and cross-race faces do not provide a clear picture of how competition for attention affects processing and memory for racial ingroups and outgroups. Examining contexts that present multiple faces of different races is important because competition between races could influence categorization and motivational processes and is also more reflective of intergroup contexts. Apparently, no research has investigated the influence of race on working memory using a paradigm where different racial stimuli compete for attention. Thus, the present research sought to investigate whether working memory for racial ingroups and outgroups is affected when different races compete for attention. In experiment 1, we used ERPs to investigate the mechanisms underlying memory differences for Black and White faces in the presence of competing racial stimuli by employing a visual short-term memory (VSTM) task adapted from Wilken and Ma (2004). The task requires individuals to attend to multiple racial faces during a brief encoding period and following a short delay, participants indicate the spatial location of one of the faces in the former array. We hypothesized that during encoding, where multiple faces of different races are presented, the race that naturally draws the person’s attention will result in better accuracy for that group; as well as, clear evidence of this attention bias in the encoding ERP. We also hypothesized that ERPs associated with motivated processing (e.g., P300) would be greater for the racial group that was better remembered later on, allowing us to examine the role of socio-cognitive factors on memory for own- and cross-race faces during competition for attention. Finally, we hypothesized that during the probe, participants would respond more accurately to the stimuli to which their attention was biased during encoding and ERP differences between correctly and incorrectly identified stimuli would be evident. In experiment 2 we sought to generalize the results from experiment 1 to other races by examining location memory for Asian and White faces. Additionally, we aimed to examine own- and cross-race effects on working memory by recruiting Asian and Caucasian participants.

## Experiment 1

### Methods

#### Participants

A previous ERP study examining working memory and racial bias (Sessa et al., 2012) obtained 16 participants. However, we used data from a behavioral study that was piloted in our lab to conduct a power analysis for the primary working memory accuracy analysis. The power analysis conducted with G^∗^Power (Faul et al., 2007) indicated that to achieve 80% power ($\eta_{p}^{2}$ = 0.21) we needed to collect data for 29 participants; thus, we aimed to analyze data for at least 30 participants. 46 students were recruited from The University of Texas at Austin. Four participants were excluded due to psychotropic medications. Additionally, data from seven participants were excluded due to problems with EEG recording (e.g., lack of EEG signal or excessive artifacts). Complete data were examined for the remaining 35 participants (22 females, 13 males, 19.49 ± 1.94 years). 25 participants were White (12 Hispanic/Latino), 7 were Asian, and 3 endorsed “other”. Participants were offered course credit or $25 for their participation. Informed consent was obtained from all participants under an IRB approved protocol at the University of Texas, Austin. Data collection was completed prior to data analysis.

#### Materials

A questionnaire was used to obtain health and demographic information. The Symbolic Racism 2000 Scale (SR2KS) was used to assess symbolic racism (Henry and Sears, 2002). In addition, the Color-Blind Racial Attitudes Scale (CoBRAS) was used to assess explicit colorblind racial attitudes (Neville et al., 2000). Stimuli for the VSTM task were selected from the Chicago Face Database (Ma et al., 2015) and altered by applying an oval mask to the face (to remove hair and clothing to the extent possible). Facial stimuli were converted to a black and white color scale to reduce variability across color images. The Chicago Face Database provides ratings for each face on a variety of measures including unusualness on a 1–7 Likert scale (1 = Not at all, 7 = Extremely). Faces rated as least unusual (lower ratings) were chosen (Black *M* = 1.86, *SD* = 0.19; White *M* = 1.76, *SD* = 0.15) to reduce confounding effects on memory. 80 face images were used including: 40 White faces (20 male and 20 female) and 40 Black faces (20 male and 20 female).

#### Procedure

Upon arrival in the lab, participants were consented and explained the procedures and then prepped for EEG recording. EEG recordings were obtained using a Biosemi electrode cap (BioSemi B.V., Amsterdam, Netherlands) with 64 active Ag/AgCl electrodes. Recording sites for the cap conformed to the 10–20 International System. Five additional channels (one below each eye, each outer canthus site, and nasion) were obtained to monitor vertical and horizontal eye movements and blinks. Channels were amplified through a Biosemi Active II amplifier system in a 24-bit DC mode at a sampling rate of 2048 Hz decimated offline to 256 Hz. Participants were seated approximately 60 cm from a 21” LED computer monitor. Participants began with a short EEG resting-state task in which they alternated between 1-min segments of eyes open and closed for eight minutes, with the order counterbalanced across participants.

**FIGURE 1:**
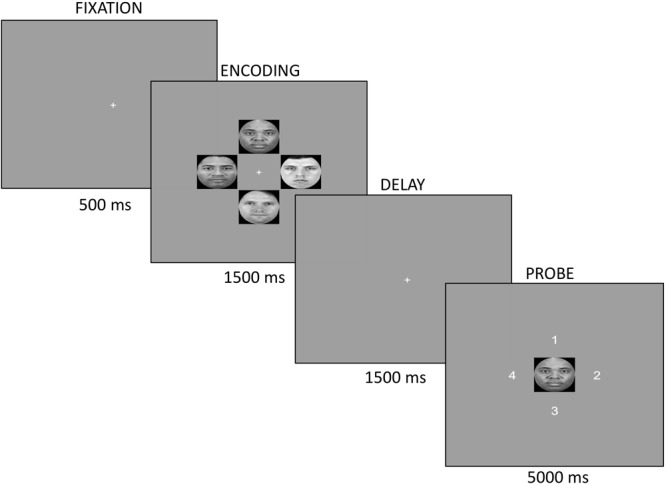
For each trial, participants fixated on a white cross for 500 ms. At encoding, four pseudo-randomly selected faces (2 Black, 2 White) were displayed in a clock-like arrangement for 1500 ms. The delay screen presented a white cross for 1500 ms. Finally, the probe screen consisted of a single face centrally located. The face probe was always one seen at encoding. Participants indicated the location of the face during encoding by pressing the button corresponding to the correct location (1, 2, 3, or 4).

Following resting EEG measures, participants completed the VSTM paradigm, which was programed using PsychoPy (Peirce, 2007). For each trial, participants viewed a fixation point for 500 ms, followed by an encoding screen. The fixation point, a white cross (height ∼0.1°), was presented in the center of the screen (0° × 0°). During encoding, four pseudo-randomly selected faces (2 Black and 2 White) placed in a clock-like arrangement were presented for 1500 ms. Face stimuli were approximately 6° (width) × 6° (height) and were presented at four equally spaced points, approximately 3.2° from the central fixation point on a gray background (Figure 1). The encoding screen was followed by a delay period containing the fixation point and lasting 1500 ms. Finally, in the probe screen a single face was presented in the center location, which was always one seen in the encoding screen. Participants were asked to indicate the location of the face during encoding by pressing one of four buttons that corresponded to the correct location (1, 2, 3, or 4). Participants were instructed to keep their eyes fixated on the central fixation point at all times. Stimuli were placed at a visual angle that would allow all faces to be visible within a typical window of attention without the need for eye movements. Male and female faces were presented in 6 separate blocks with block order counterbalanced across participants. To familiarize participants with the task, they completed 12 practice trials after which they had the opportunity to ask for further clarification of instructions. Facial stimuli for the practice trials were drawn from the same sample that was used for the experimental trials. Following practice, participants continued the task where a total of 360 experimental trials were presented across the six blocks with a short break after each block. Throughout the experimental trials, each face was presented an average of 18 times (*SD* = 4.04). Afterward, participants completed five questionnaires in the following order: CES-D, demographic questionnaire, contact questionnaire, SR2KS, and CoBRAS. Participants were debriefed at the end of the experiment.

#### Behavioral Data Reduction

Behavioral data analysis was performed using R 3.3.2 (R Core Team, 2016). Trials with false starts (reaction times below 300 ms) or with reaction times greater than 2.5 standard deviations above the participant’s mean reaction time were identified and excluded. Overall, 2.89% of the data were excluded.

#### EEG Data Processing

Continuous data were imported off-line and data analysis was conducted using BrainVision Analyzer 2.0 (BrainVision LLC, Morrisville, NC, United States). Data were re-referenced to the average of the left and right mastoids. However, to examine the N170 component, data were re-referenced to the average of all EEG scalp sites based on previous work by Joyce and Rossion (2005) on the best approach for examining the N170 (see also Zhou et al., 2015b). A Butterworth Zero Phase Filter with a low cutoff of 0.1 Hz (12 dB/oct) and a high cutoff of 40 Hz (48 dB/oct) was applied to filter EEG data. Channels to monitor eye-movement artifacts were computed offline. The horizontal EOG channel was created using a bipolar montage of the nasion electrode and each outer canthi electrode (HEOG). For the vertical EOG channel, a bipolar montage of the FP1 and FP2 channels and the electrode placed on the corresponding inferior orbit were used (VEOG). Independent Components Analysis (ICA) was applied to identify and reject ocular artifacts using a meaned slope algorithm for blink detection with the left VEOG as reference. Remaining artifacts were identified using a semiautomatic inspection approach resulting in less than 2% of the data excluded per person. Overall, an average of 0.35% ± 0.003 of the data were excluded. Bad EEG channels were interpolated using a spline interpolation (order: 4, degree: 10, lambda: 1E-05) for three participants, with only one or two channels interpolated for each participant.

Following preprocessing, EEG data were epoched from -200 to 1500 ms post-stimulus onset for two time windows – encoding and probe presentations. For both periods, ERPs were generated by averaging epochs based on the race of the face (Black and White) during the probe period and the accuracy of the location response (correct and incorrect) and were baseline corrected to an average of the -200-0 ms prestimulus interval. Visual inspection and previous work guided examination of several ERP components of interest: the N170, N200, P300, and late positive component (LPC). The scalp locations used to measure each component were visually estimated from projecting the grand-average ERPs over the entire scalp. The time window for the N170, N200, and P300 was obtained by identifying the peak latency ± 25 ms. N170 amplitudes from 145 to 195 and 135 to 185 ms post-stimulus onset for encoding and probe respectively, were averaged across the left (P7, PO7, P9) and right (P8, PO8, P10) parietal scalp sites separately (see Stahl et al., 2008; Cassidy et al., 2014). For the encoding N200, amplitudes from 260 to 310 ms post-stimulus onset were averaged across 3 scalp locations – Fz, Cz, and Pz scalp sites (see Ito et al., 2004; Wiese, 2012). P300 amplitudes from 325 to 375 ms and 350 to 400 ms post-stimulus onset for encoding and probe respectively, were averaged across 3 scalp locations – frontal (Fz, F1, F2), central (Cz, C1, C2), and parietal (Pz, P1, P2) scalp sites. Previous research indicated that the LPC is a separate component from the P300 (Foti et al., 2009); therefore, LPC amplitudes from 500 to 800 ms, a time window that did not overlap with the P300, were averaged across 3 scalp locations - frontal (Fz, F1, F2), central (Cz, C1, C2), and parietal (Pz, P1, P2) scalp sites. Mean amplitudes were generated and entered into a repeated measures ANOVA using R 3.3.2 (R Core Team, 2016). We report all measures, manipulations and exclusions for this study.

### Results

#### Behavioral Results

**FIGURE 2:**
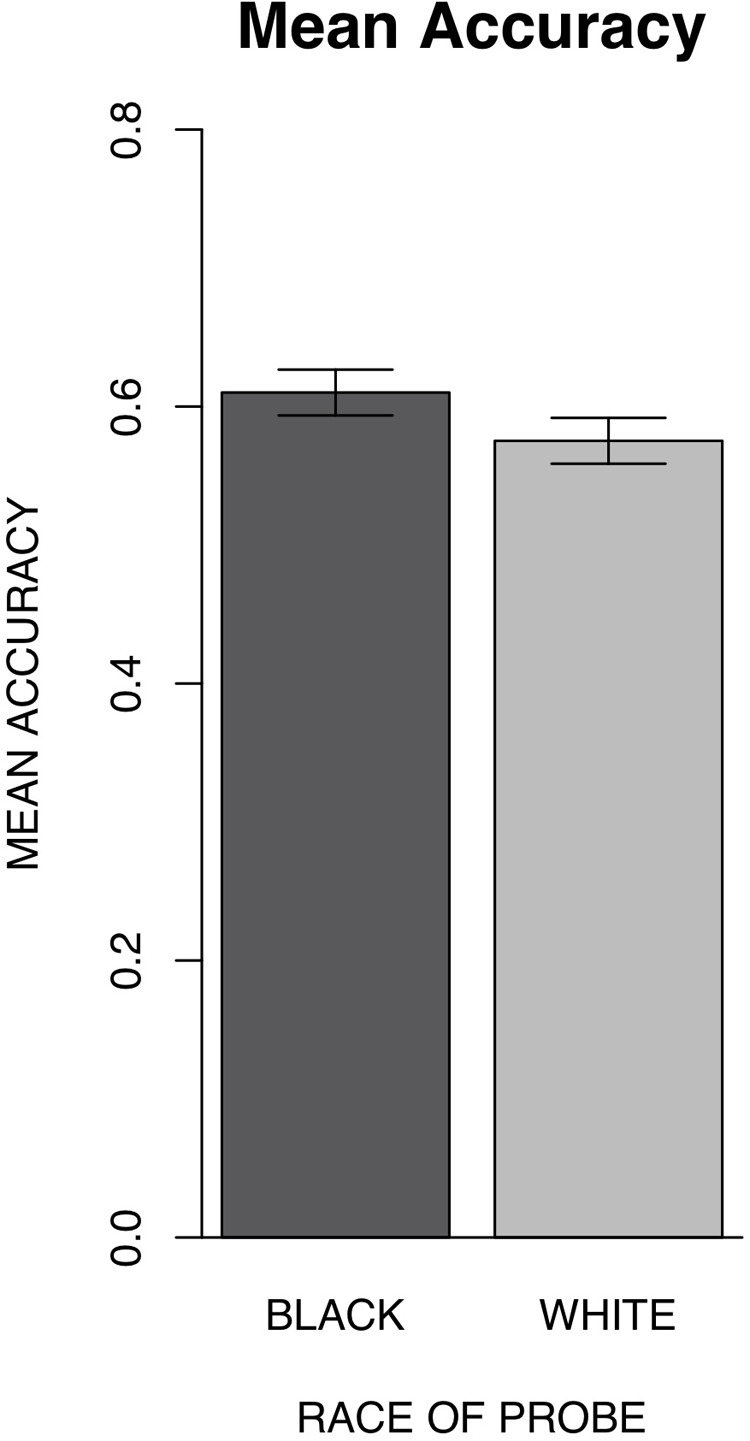
Experiment 1 mean accuracy for race of probe along with 95% confidence intervals. Results indicated significantly better location accuracy for Black (*M* = 0.61, *SD* = ± 0.05) than White (*M* = 0.58, *SD* = ± 0.05) probes.

A one-way repeated measures ANOVA with race (Black vs. White) as a within-subjects factor was conducted to examine accuracy differences between Black and White probe trials and indicated significantly greater accuracy for the location judgment of Black faces (*M* = 0.61, *SD* = ± 0.05), relative to White faces (*M* = 0.58, *SD* = ± 0.05), [*F*(1,34) = 9.16, *p* = 0.005, $\eta_{p}^{2}$ = 0.21] (Figure 2). Reaction times for correct trials were also examined in a one-way repeated measures ANOVA with race (Black vs. White) as a within-subjects factor and indicated no significant differences in reaction times between Black and White probe trials [*F*(1,34) = 1.00, *p* = 0.33, $\eta_{p}^{2}$ = 0.03].

**FIGURE 3:**
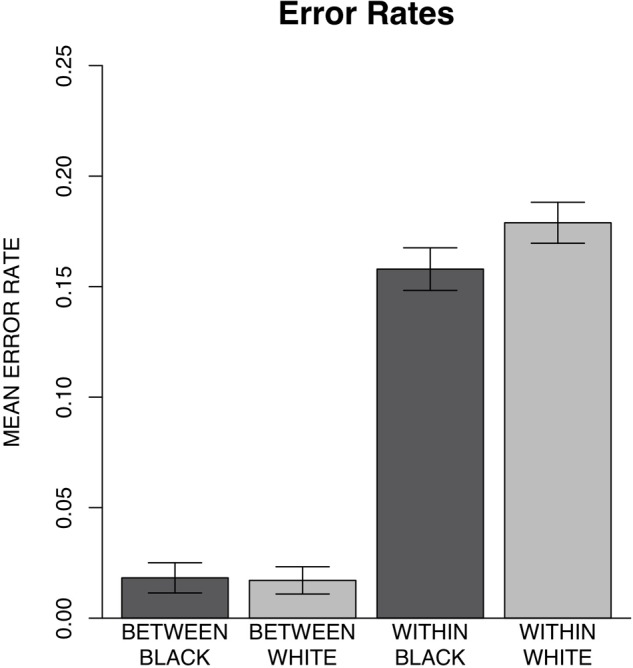
Experiment 1 mean corrected error rate per type and probe race along with 95% confidence intervals. Results indicated significantly more within-race errors for White (*M* = 0.18, *SD* = ± 0.03) than Black faces (*M* = 0.16, *SD* = ± 0.03) but no significant differences in between-race errors for White (*M* = 0.03, *SD* = ± 0.02) and Black faces (*M* = 0.04, *SD* = ± 0.02).

In the present task, error rates were corrected since chance alone would result in a higher number of between-race than within-race errors. That is, while two responses would result in between-race errors, only one response would result in a within-race error. Thus, to make the number of between-race and within-race errors directly comparable, each subject’s between-race error rate was reduced by one-half prior to data analysis, as done in previous research (Taylor et al., 1978). A 2 × 2 repeated measures ANOVA with type of error (within-race vs. between-race) and probe race (Black vs. White) as within-subjects factors was conducted to examine the types of errors made by participants. There was a significant interaction between error type and probe race [*F*(1,34) = 14.40, *p* < 0.001, $\eta_{p}^{2}$ = 0.30]. *Post hoc* Bonferroni comparisons revealed significantly more within-race errors for White (*M* = 0.18, *SD* = ± 0.03) than Black faces (*M* = 0.16, *SD* = ± 0.03) [*t*(63.59) = -4.89, *p* < 0.001, *d* = -0.38]. However, between-race errors for White (*M* = 0.03, *SD* = ± 0.02) and Black faces (*M* = 0.04, *SD* = ± 0.02) were not significantly different [*t*(63.59) = 0.27, *p* = 0.79, *d* = 0.07]. There was also a significant main effect of error type, participants made more within-race errors than between-race errors [*F*(1,34) = 772.82, *p* < 0.001, $\eta_{p}^{2}$ = 0.96] and a significant main effect of probe race with more errors for White than Black probes [*F*(1,34) = 9.90, *p* = 0.003, $\eta_{p}^{2}$ = 0.23] (Figure 3).

We also examined the relationship between accuracy and explicit measures of racial attitudes. First, we calculated an accuracy difference score by subtracting accuracy for White faces from accuracy for Black faces (Black accuracy – White accuracy). Subsequently, the difference score was correlated to the overall SR2KS and the CoBRAS scores. Since the Contact Questionnaire, which examined contact with other races, was added to the experiment after data collection had started, responses were not obtained for all participants and therefore data on the Contact Questionnaire was not analyzed. No significant correlations were found between the accuracy difference score and the SR2KS score nor the CoBRAS score. The relationship between error rates and explicit measures of racial prejudice was also examined. An error rate difference score was calculated by subtracting within-race errors from between-race errors (between-race errors – within-race errors). No significant correlations were found between the error rate difference score and the SR2KS nor the CoBRAS.

#### ERP Results

##### Encoding

Tables 1a,b show the average amplitudes for the N170, N200, P300 and LPC during encoding for each condition (defined by probe accuracy) across the scalp locations tested. The N170 at encoding was examined by testing the mean amplitude for 145–195 ms post-stimulus (presentation of four faces) onset across the 2 scalp locations in a 2 × 2 × 2 ANOVA with race (Black vs. White), accuracy (correct vs. incorrect), and location (left vs. right) as within-subjects factors. Results indicated that the three-way interaction between race, accuracy, and location was not significant [*F*(1,34) = 0.80, *p* = 0.38, $\eta_{p}^{2}$ = 0.02]. Additionally, there were no significant interactions between race and location [*F*(1,34) = 0.54, *p* = 0.47, $\eta_{p}^{2}$ = 0.02], accuracy and location [*F*(1,34) = 1.00, *p* = 0.32, $\eta_{p}^{2}$ = 0.03], or race and accuracy [*F*(1,34) = 2.85, *p* = 0.10, $\eta_{p}^{2}$ = 0.08]. There were also no significant main effects of race [*F*(1,34) = 3.36, *p* = 0.08, $\eta_{p}^{2}$ = 0.09], accuracy [*F*(1,34) = 3.22, *p* = 0.08, $\eta_{p}^{2}$ = 0.09], or hemisphere [*F*(1,34) = 3.86, *p* = 0.06, $\eta_{p}^{2}$ = 0.10] on N170 amplitudes.

**Table 1:** Mean ERP amplitudes (mV) for encoding period.

	N170		N200		
	Left hemisphere	Right hemisphere	Fz	Cz	Pz
		
Condition	*M*(*SD*)	*M*(*SD*)	*M*(*SD*)	*M*(*SD*)	*M*(*SD*)
Black correct	0.28 (0.61)	–0.56 (0.79)	–3.87 (1.25)	–3.39 (1.06)	0.08 (0.69)
White correct	0.26 (0.50)	–0.61 (0.66)	–3.47 (1.25)	–3.13 (1.00)	–0.09 (0.87)
Black incorrect	0.29 (0.79)	–0.57 (0.88)	–3.36 (1.06)	–3.32 (1.41)	–0.11 (0.98)
White incorrect	–0.22 (0.90)	–0.83 (0.80)	–3.31 (1.27)	–2.97 (0.91)	0.27 (0.84)

**Table 1b:** Mean ERP amplitudes (mV) for encoding period.

	P300			LPC		
	Frontal	Central	Parietal	Frontal	Central	Parietal
		
Condition	*M*(*SD*)	*M*(*SD*)	*M*(*SD*)	*M*(*SD*)	*M*(*SD*)	*M*(*SD*)
Black correct	–1.98 (1.26)	–1.48 (1.07)	1.34 (0.70)	–1.65 (1.13)	–1.22 (1.12)	0.24 (0.83)
White correct	–1.58 (1.24)	–1.44 (1.20)	0.97 (0.83)	–1.68 (1.01)	–1.42 (1.01)	–0.05 (0.86)
Black incorrect	–1.45 (1.28)	–1.42 (1.30)	0.96 (0.94)	–1.19 (1.17)	–1.27 (1.37)	–0.13 (0.95)
White incorrect	–1.31 (1.01)	–1.03 (0.96)	1.33 (0.82)	–1.30 (1.02)	–1.11 (1.03)	0.32 (0.86)

The N200 at encoding was examined by testing the mean amplitude for 260–310 ms post-stimulus onset in a 2 × 2 ANOVA for each scalp location (Fz, Cz, and Pz) with race (Black vs. White) and accuracy (correct vs. incorrect) as within-subjects factors. For the Fz location, there was no significant interaction between race and accuracy [*F*(1,34) = 0.92, *p* = 0.34, $\eta_{p}^{2}$ = 0.03], and no significant main effects of race [*F*(1,34) = 1.36, *p* = 0.25, $\eta_{p}^{2}$ = 0.04], or accuracy [*F*(1,34) = 2.12, *p* = 0.06, $\eta_{p}^{2}$ = 0.15]. Secondly, for the Cz location, there was no significant interaction between race and accuracy [*F*(1,34) = 0.08, *p* = 0.78, $\eta_{p}^{2}$ = 0.002], and no significant main effects of race [*F*(1,34) = 2.66, *p* = 0.11, $\eta_{p}^{2}$ = 0.07], or accuracy [*F*(1,34) = 0.31, *p* = 0.58, $\eta_{p}^{2}$ = 0.009]. Finally, for the Fz location, there no significant interaction between race and accuracy [*F*(1,34) = 3.82, *p* = 0.06, $\eta_{p}^{2}$ = 0.10]. Moreover, there were no significant main effects of race [*F*(1,34) = 0.50, *p* = 0.48, $\eta_{p}^{2}$ = 0.01], or accuracy [*F*(1,34) = 0.38, *p* = 0.54, $\eta_{p}^{2}$ = 0.01].

**FIGURE 4:**
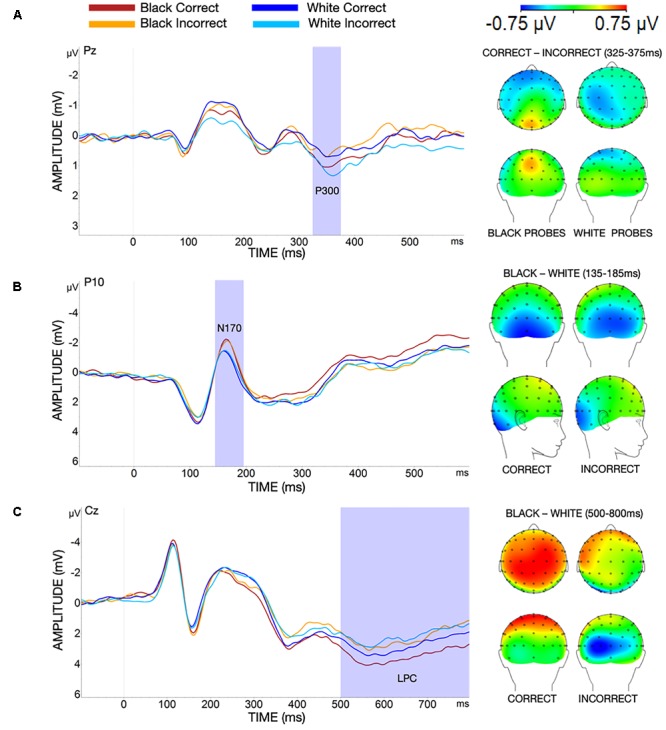
**(A)** Encoding: P300 at site Pz. More positive P300s were elicited by Black correct and White incorrect trials than Black incorrect and White correct trials. Scalp map shows the P300 differences between correct and incorrect trials for Black (left) and White (right) probes. **(B)** Probe: N170 at site P10. More negative N170s were elicited for Black than White probes. Scalp map shows the N170 differences between Black and White trials for correct (left) and incorrect (right) trials. **(C)** Probe: LPC at site Cz. More positive LPC amplitudes were observed for correct than incorrect trials and for Black than White faces. Scalp map shows the LPC differences between Black and White trials for correct (left) and incorrect (right) trials.

To examine the P300 at encoding (Figure 4A) we tested the mean amplitude for 325–375 ms post-stimulus onset in a 2 × 2 ANOVA for each scalp location (frontal, central, and parietal) with race (Black vs. White) and accuracy (correct vs. incorrect) as within-subjects factors. Results for the frontal location did not show a significant interaction between race and accuracy [*F*(1,34) = 1.27, *p* = 0.50, $\eta_{p}^{2}$ = 0.01], or a significant main effect of race [*F*(1,34) = 1.41, *p* = 0.24, $\eta_{p}^{2}$ = 0.04]. However, there was a significant main effect of accuracy [*F*(1,34) = 4.53, *p* = 0.04, $\eta_{p}^{2}$ = 0.12], driven by more positive P300 amplitudes for incorrect than correct trials. P300 amplitudes for the central location did not show a significant interaction between race and accuracy [*F*(1,34) = 0.81, *p* = 0.37, $\eta_{p}^{2}$ = 0.02]. There were also no significant main effects of race [*F*(1,34) = 1.37, *p* = 0.25, $\eta_{p}^{2}$ = 0.04] or accuracy [*F*(1,34) = 1.41, *p* = 0.24, $\eta_{p}^{2}$ = 0.04] for central P300 amplitudes. Finally, results for the parietal location indicated a significant interaction between race and accuracy [*F*(1,34) = 7.30, *p* = 0.01, $\eta_{p}^{2}$ = 0.18]. *Post hoc* Bonferroni comparisons were conducted to examine the nature of the interaction but revealed no significant differences between parietal P300s at encoding for Black than White probe trials when people responded correctly to the location probe [*t*(67.99) = 1.93, *p* = 0.06, *d* = 0.11]. Likewise, the reverse pattern, greater parietal P300s at encoding for White than Black probe trials when people responded incorrectly to the location probe, was not significant [*t*(67.99) = -1.91, *p* = 0.06, *d* = -0.11]. The interaction between race and accuracy for the parietal location suggests that by the time window captured in the P300, there is greater attention to Black faces (outgroup – related to better accuracy); however, these results must be interpreted with caution given that *post hoc* comparisons did not indicate significant differences between Black and White probe trials for correct and incorrect answers.^1^

A final component examined at encoding was the LPC. The mean amplitude for 500–800 ms post-stimulus onset was tested in a 2 × 2 ANOVA for each scalp location (frontal, central, and parietal) with race (Black vs. White) and accuracy (correct vs. incorrect) as within-subjects factors. Results for the frontal location did not show a significant interaction between race and accuracy [*F*(1,34) = 0.07, *p* = 0.79, $\eta_{p}^{2}$ = 0.002], or a significant main effect of race [*F*(1,34) = 0.10, *p* = 0.76, $\eta_{p}^{2}$ = 0.003]. However, there was a significant main effect of accuracy [*F*(1,34) = 5.55, *p* = 0.02, $\eta_{p}^{2}$ = 0.14] on LPC amplitudes for the frontal location, with more positive amplitudes for incorrect than correct trials. Results for the central location did not show a significant interaction between race and accuracy [*F*(1,34) = 0.66, *p* = 0.42, $\eta_{p}^{2}$ = 0.02], or significant main effects of race [*F*(1,34) = 0.02, *p* = 0.90, $\eta_{p}^{2}$ < 0.001], or accuracy [*F*(1,34) = 0.54, *p* = 0.47, $\eta_{p}^{2}$ = 0.02]. Finally, results for the parietal location revealed a significant interaction between race and accuracy [*F*(1,34) = 8.28, *p* = 0.007, $\eta_{p}^{2}$ = 0.20]. *Post hoc* Bonferroni comparisons revealed a significant difference in LPC amplitudes driven by more positive parietal LPCs at encoding for White than Black probe trials when people responded incorrectly [*t*(62.63) = -2.08, *p* = 0.04, *d* = -0.14]. However, there was no significant difference in parietal LPC amplitudes between White and Black probe trials when people responded correctly, [*t*(62.63) = 1.34, *p* = 0.19, *d* = 0.10].

##### Probe

ERPs were analyzed across the probe period for the N170, P300, and LPC components. Tables 2a,b show the mean ERP amplitudes during the probe period for each condition across all locations. Data for the N170 (average amplitude for 135–185 ms post-stimulus onset; Figure 4B) during presentation of the single probe face were examined across the left and right hemispheres in a 2 × 2 × 2 ANOVA with race (Black vs. White), accuracy (correct vs. incorrect), and hemisphere (left vs. right) as within-subjects factors. Results indicated that the three-way interaction between race, accuracy, and location was not significant [*F*(1,34) = 0.14, *p* = 0.71, $\eta_{p}^{2}$ = 0.004]. Additionally, there were no significant interactions between race and location [*F*(1,34) = 0.42, *p* = 0.52, η_*p*_^2^ = 0.01], accuracy and location [*F*(1,34) = 1.72, *p* = 0.20, $\eta_{p}^{2}$ = 0.05], nor race and accuracy [*F*(1,34) = 0.11, *p* = 0.74, $\eta_{p}^{2}$ = 0.003]. There was no significant main effect of accuracy [*F*(1,34) < 0.01, *p* = 0.99, $\eta_{p}^{2}$ < 0.001]. However, results indicated a significant main effect of hemisphere [*F*(1,34) = 8.10, *p* = 0.007, $\eta_{p}^{2}$ = 0.19], with more negative N170 amplitudes in the left relative to the right hemisphere. Additionally, there was a significant main effect of race [*F*(1,34) = 12.08, *p* = 0.001, $\eta_{p}^{2}$ = 0.26], with greater N170 amplitudes for Black than White faces.^2^

**Table 2a:** Mean ERP amplitudes (mV) for probe period.

	N170		P300		
	Left hemisphere	Right hemisphere	Frontal	Central	Parietal
		
Condition	*M*(*SD*)	*M*(*SD*)	*M*(*SD*)	*M*(*SD*)	*M*(*SD*)
Black correct	–1.10 (0.72)	–0.15 (0.80)	0.56 (1.31)	2.78 (1.11)	5.31 (0.89)
White correct	–0.77 (0.65)	0.22 (0.74)	0.46 (1.10)	2.43 (1.11)	5.07 (0.87)
Black incorrect	–1.19 (0.65)	–0.11 (0.63)	–0.22 (1.43)	1.98 (1.23)	4.73 (1.07)
White incorrect	–0.86 (0.53)	0.36 (0.53)	–0.47 (1.22)	1.85 (1.13)	4.70 (0.93)

**Table 2b:** Mean ERP amplitudes (mV) for probe period.

	LPC		
	Frontal	Central	Parietal
Condition	*M*(*SD*)	*M*(*SD*)	*M*(*SD*)
Black correct	0.11 (1.11)	3.39 (0.93)	4.40 (0.71)
White correct	–0.46 (1.00)	2.59 (1.07)	3.86 (0.80)
Black incorrect	–0.90 (1.19)	2.18 (1.14)	3.43 (1.15)
White incorrect	–1.25 (1.20)	1.94 (0.95)	3.27 (0.92)

Next, the P300 at probe was examined (average amplitude for 350–400 ms post-stimulus onset) in a 2 × 2 ANOVA for each scalp location (frontal, central, and parietal) with race (Black vs. White) and accuracy (correct vs. incorrect) as within-subjects factors. Results for the frontal location did not show a significant interaction between race and accuracy [*F*(1,34) = 0.14, *p* = 0.71, $\eta_{p}^{2}$ = 0.004] nor a significant main effect of race [*F*(1,34) = 0.47, *p* = 0.50, $\eta_{p}^{2}$ = 0.02]. However, results indicated a significant main effect of accuracy [*F*(1,34) = 18.64, *p* < 001, $\eta_{p}^{2}$ = 0.35], with greater P300s for correct than incorrect responses in the frontal location. P300 amplitude results for the central location did not show a significant interaction between race and accuracy [*F*(1,34) = 0.52, *p* = 0.47, $\eta_{p}^{2}$ = 0.02], or a significant main effect of race [*F*(1,34) = 0.98, *p* = 0.33, $\eta_{p}^{2}$ = 0.03]. However, there was a significant main effect of accuracy [*F*(1,34) = 15.69, *p* < 0.001, $\eta_{p}^{2}$ = 0.32] with greater P300 amplitudes for correct than incorrect responses in the central location. Finally, for the parietal location there was no significant interaction between race and accuracy [*F*(1,34) = 0.47, *p* = 0.50, $\eta_{p}^{2}$ = 0.01], or a significant main effect of race [*F*(1,34) = 0.51, *p* = 0.48, $\eta_{p}^{2}$ = 0.01] on P300 amplitudes. However, similar to the frontal and central locations, results for the parietal location showed a significant main effect of accuracy [*F*(1,34) = 11.56, *p* = 0.002, $\eta_{p}^{2}$ = 0.25], with more positive P300 amplitudes for correct than incorrect responses.

Finally, the late positive component (LPC) at probe was analyzed (average amplitude for 500–800 ms post-stimulus onset; Figure 4C) in a 2 × 2 ANOVA for each scalp location (frontal, central, and parietal) with race (Black vs. White) and accuracy (correct vs. incorrect) as within-subjects factors. For the frontal location, there was no significant interaction between race and accuracy [*F*(1,34) = 0.37, *p* = 0.55, $\eta_{p}^{2}$ = 0.01] on LPC amplitudes. However, there was a significant main effect of race [*F*(1,34) = 4.72, *p* = 0.04, $\eta_{p}^{2}$ = 0.12], with greater frontal LPC amplitudes for Black than White probes. There was also a significant main effect of accuracy [*F*(1,34) = 24.07, *p* < 0.001, $\eta_{p}^{2}$ = 0.41], with greater frontal LPCs for correct than incorrect responses. LPC amplitude results for the central location did not show a significant interaction between race and accuracy [*F*(1,34) = 3.62, *p* = 0.07, η_p_^2^ = 0.10]. However, there was a significant main effect of race [*F*(1,34) = 6.25, *p* = 0.02, η_p_^2^ = 0.16], with greater central LPCs for Black than White probes. Additionally, there was a significant main effect of accuracy [*F*(1,34) = 35.75, *p* < 0.001, η_p_^2^ = 0.51], with greater central LPCs for correct than incorrect responses. Results for the parietal location did not indicate a significant interaction between race and accuracy [*F*(1,34) = 1.42, *p* = 0.24, η_p_^2^ = 0.04] on LPC amplitudes. There was no significant main effect of race [*F*(1,34) = 4.32, *p* = 0.05, η_p_^2^ = 0.11] on parietal LPC amplitudes. Lastly, results indicated a significant main effect of accuracy [*F*(1,34) = 35.51, *p* < 0.001, η_p_^2^ = 0.51], with greater parietal LPCs for correct than incorrect responses.

##### ERP and Explicit Measures Results

To examine the relationship between ERPs and explicit racial bias measures, an amplitude difference score was calculated by subtracting the amplitude for White correct trials from the amplitude for Black correct trials (Black correct amplitude – White correct amplitude). The difference score served as an index of attentional bias. Analyses were conducted only for the ERP components and locations for which there was a significant main effect of race or a race × accuracy interaction for the encoding or probe periods. After calculating the amplitude difference score, the ERP difference score was correlated to the overall scores on the SR2KS and CoBRAS. After correcting for multiple comparisons, there were no significant correlations between SR2KS scores and the encoding P300 (*r* = -0.19, *p* > 0.25), and LPC (*r* = -0.05, *p* > 0.25) at parietal locations. There were also no significant correlations between CoBRAS scores and the encoding P300 (*r* = -0.18, *p* > 0.25), and LPC (*r* = -0.06, *p* > 0.25) at parietal locations. For the probe period, there were no significant correlations between SR2KS scores and the left hemisphere N170 (*r* = -0.16, *p* > 0.25), the right hemisphere N170 (*r* = -0.31, *p* > 0.25), the frontal LPC (*r* = 0.12, *p* > 0.25), and the central LPC (*r* = 0.13, *p* > 0.25). Finally, for the probe period there were also no significant correlations between CoBRAS scores and the left hemisphere N170 (*r* = -0.08, *p* > 0.25), the right hemisphere N170 (*r* = -0.31, *p* > 0.25), the frontal LPC (*r* = -0.05, *p* > 0.25), and the central LPC (*r* = -0.05, *p* > 0.25).

### Discussion

In experiment 1, participants had more accurate working memory for Black than White faces during competition for attention between races; a finding inconsistent with the own-race bias (Meissner and Brigham, 2001; Walker and Hewstone, 2008; Wiese et al., 2014). One explanation for the observed results is that darker faces resulted in greater initial attentional capture and subsequently better memory. Thus, the effects could be attributed to skin color differences rather than race *per se*. However, this seems unlikely since the early ERP components during encoding (i.e., N170 and N200) did not indicate differences in initial attention to Black and White faces. Nevertheless, a second experiment was conducted to examine whether the prior findings were driven by differences in skin color rather than race.

## Experiment 2

### Introduction

The primary goal of experiment 2 was to determine whether the greater location accuracy for Black than White faces resulted from differences in skin color. Thus, we examined memory for Asian and White faces since skin color differences between these races tend to be less pronounced. We also examined own- and cross-race effects in Asian and Caucasian participants. We hypothesized that Caucasian participants would have better memory for Asian than White faces whereas Asian participants would have better memory for White than Asian faces.

### Methods

#### Participants

151 students were recruited from The University of Texas at Austin but data was only examined for participants who indicated they were Caucasian or Asian (*n* = 105). Of those participants, six were excluded due to data recording problems or below-chance accuracy. Hence, complete data were examined for 61 Caucasian participants (38 females, 22 males, 1 “other”, 19.86 ± 6.52 years) and 38 Asian participants (21 females, 16 males, 1 “other”, 18.71 ± 0.96 years). Participants were offered course credit for their participation. Informed consent was obtained from all participants under an IRB approved protocol at the University of Texas, Austin. Data collection was completed prior to data analysis.

#### Materials

The questionnaire and materials used for this experiment were the same as Experiment 1, with the exception that the stimuli used included White and Asian faces from the Chicago Face Database (Ma et al., 2015). 80 face images were used including: 40 White faces (20 male and 20 female) and 40 Asian faces (20 male and 20 female).

#### Procedure

**FIGURE 5:**
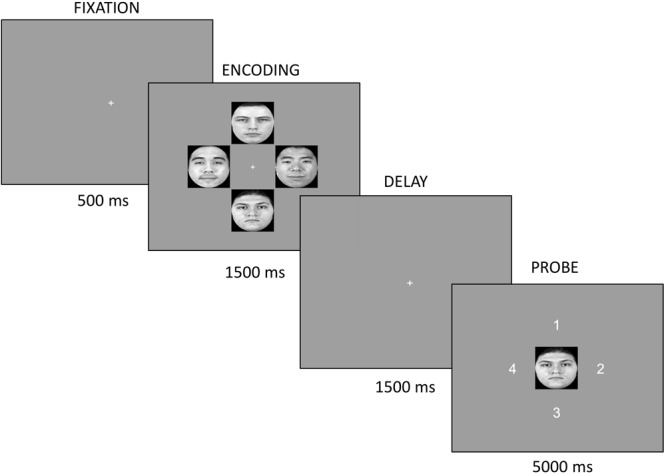
For each trial, participants fixated on a white cross for 500 ms. At encoding, four pseudo-randomly selected faces (2 Asian, 2 White) were displayed in a clock-like arrangement for 1500 ms. The delay screen presented a white cross for 1500 ms. Finally, the probe screen consisted of a single face centrally located. The face probe was always one seen at encoding. Participants indicated the location of the face during encoding by pressing the button corresponding to the correct location (1, 2, 3, or 4).

The procedure for this experiment was the same as Experiment 1 with the exception of the following: EEG recordings were not obtained while participants completed the VSTM paradigm, and during encoding participants viewed two Asian and two White faces (Figure 5).

#### Behavioral Data Reduction

Behavioral data analysis was the same as Experiment 1. Overall, 4.09% of the data were excluded for Caucasian participants, and 3.93% of the data were excluded for Asian participants.

### Results

**FIGURE 6:**
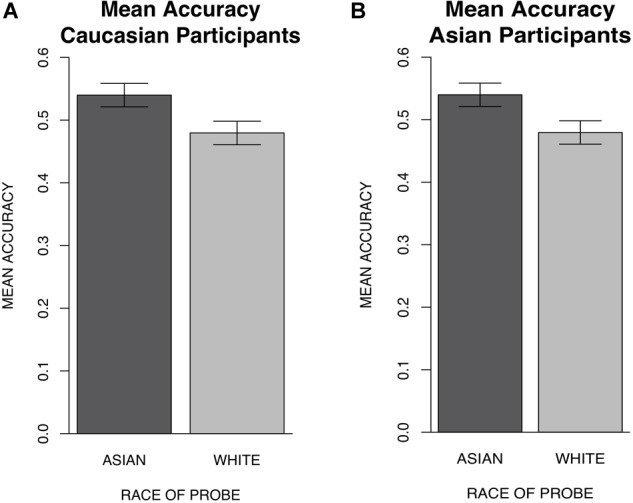
Experiment 2 mean accuracy for race of probe along with 95% confidence intervals for Caucasian and Asian participants. **(A)** Results for Caucasian participants indicated significantly better location accuracy for Asian (*M* = 0.49, *SD* = ± 0.04) than White (*M* = 0.44, *SD* = ± 0.04) probes. **(B)** Results for Asian participants indicated significantly better location accuracy for Asian (*M* = 0.54, *SD* = ± 0.06) than White (*M* = 0.48, *SD* = ± 0.06) probes.

A one-way repeated measures ANOVA with race (Asian vs. White) as a within-subjects factor was conducted separately for Caucasian participants and Asian participants to examine accuracy differences between Asian and White probe trials. ANOVA results for Caucasian participants indicated significantly greater accuracy for the location judgment of Asian faces (*M* = 0.49, *SD* = ± 0.04), relative to White faces (*M* = 0.44, *SD* = ± 0.04), [*F*(1,60) = 41.88, *p* < 0.001, $\eta_{p}^{2}$ = 0.41] (Figure 6A). Results for Asian participants also indicated significantly greater accuracy for the location judgment of Asian faces (*M* = 0.54, *SD* = ± 0.06), relative to White faces (*M* = 0.48, *SD* = ± 0.06), [*F*(1,37) = 21.37, *p* < 0.001, $\eta_{p}^{2}$ = 0.73] (Figure 6B). Reaction times for correct trials were also examined for Caucasian and Asian participants in separate one-way repeated measures ANOVAs with race (Black vs. White) as a within-subjects factor. Results for Caucasian participants indicated significantly faster reaction times for the location judgment of Asian faces (*M* = 0.99, *SD* = ± 0.03) than White faces (*M* = 1.00, *SD* = ± 0.03) [*F*(1,60) = 7.81, *p* = 0.007, $\eta_{p}^{2}$ = 0.12]. Additionally, results for Asian participants indicated significantly faster reaction times for the location judgment of Asian faces (*M* = 0.91, *SD* = ± 0.03) than White faces (*M* = 0.92, *SD* = ± 0.03) [*F*(1,37) = 4.43, *p* = 0.04, $\eta_{p}^{2}$ = 0.11]. In contrast with the reaction time results from experiment 1 indicating no differences in reaction times for Black and White faces, the results from experiment 2 suggest an earlier response to Asian faces. It is possible that the earlier response to Asian faces contributed to better working memory accuracy for Asian faces; however, future work will have to examine this possibility since ERPs were not obtained in the present experiment.

**FIGURE 7:**
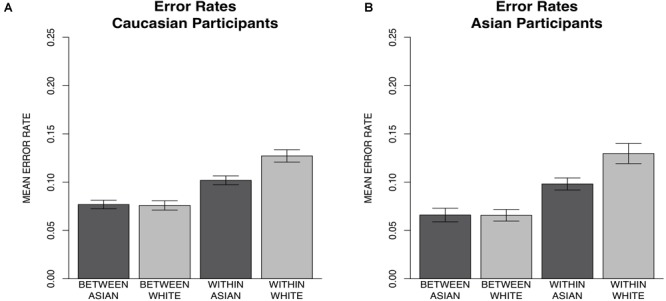
Experiment 2 mean corrected error rate per type and probe race along with 95% confidence intervals for Caucasian and Asian participants. **(A)** Caucasian participants made significantly more within-race errors for White (*M* = 0.13, *SD* = ± 0.03) than Asian (*M* = 0.10, *SD* = ± 0.03) faces but did not differ in between-race errors for White (*M* = 0.08, *SD* = ± 0.02) and Asian (*M* = 0.08, *SD* = ± 0.02) faces. **(B)** Asian participants made significantly more within-race errors for White (*M* = 0.13, *SD* = ± 0.03) than Asian (*M* = 0.10, *SD* = ± 0.02) faces but did not differ in between-race errors for White (*M* = 0.07, *SD* = ± 0.02) and Asian (*M* = 0.07, *SD* = ± 0.02) faces.

Like in experiment 1, error rates were corrected since chance alone would result in a higher number of between-race than within-race errors. Separate 2 × 2 repeated measures ANOVAs with type of error (within-race vs. between-race) and probe race (Asian vs. White) as within-subjects factors were conducted for Caucasian participants and Asian participants to examine the types of errors made. For Caucasian participants, there was a significant interaction between error type and probe race [*F*(1,60) = 38.41, *p* < 0.001, $\eta_{p}^{2}$ = 0.39). *Post hoc* Bonferroni comparisons revealed significantly more within-race errors for White (*M* = 0.13, *SD* = ± 0.03) than Asian faces (*M* = 0.10, *SD* = ± 0.03) [*t*(118.32) = -8.92, *p* < 0.001, *d* = -0.64]. However, between-race errors for White (*M* = 0.08, *SD* = ± 0.02) and Asian faces (*M* = 0.08, *SD* = ± 0.02) were not significantly different [*t*(118.32) = 0.35, *p* = 0.73, *d* = 0.04]. There was also a significant main effect of error type, participants made more within-race errors than between-race errors [*F*(1,60) = 125.16, *p* < 0.001, $\eta_{p}^{2}$ = 0.68] and a significant main effect of probe race with more errors for White than Asian probes [*F*(1,60) = 41.73, *p* < 0.001, $\eta_{p}^{2}$ = 0.41] (Figure 7A). Results for Asian participants also indicated a significant interaction between error type and probe race [*F*(1,37) = 30.09, *p* < 0.001, $\eta_{p}^{2}$ = 0.45]. *Post hoc* Bonferroni comparisons revealed significantly more within-race errors for White (*M* = 0.13, *SD* = ± 0.03) than Asian faces (*M* = 0.10, *SD* = ± 0.02) [*t*(71.93) = -7.00, *p* < 0.001, *d* = -1.01]. However, between-race errors for White (*M* = 0.07, *SD* = ± 0.02) and Asian faces (*M* = 0.07, *SD* = ± 0.02) were not significantly different [*t*(71.93) = 0.07, *p* = 0.95, *d* = 0.01]. There was also a significant main effect of error type, participants made more within-race errors than between-race errors [*F*(1,37) = 102.20, *p* < 0.001, $\eta_{p}^{2}$ = 0.73] and a significant main effect of probe race with more errors for White than Asian probes [*F*(1,37) = 20.57, *p* < 0.001, $\eta_{p}^{2}$ = 0.36] (Figure 7B).

### Discussion

Results from experiment 2 indicated that both Caucasian and Asian participants had better memory for Asian than White faces, indicating that the effects from experiment 1 were not solely a result of skin color differences between Black and White faces. However, contrary to our initial hypothesis, both Caucasian and Asian participants had better location memory for Asian relative to White faces, we will return to this issue in the general discussion.

## General Discussion

The present research investigated the neurocognitive mechanisms associated with racial bias during competition for attention to provide a closer representation of intergroup interactions. In experiment 1, we independently examined distinct processing stages and their brain electrophysiological response by combining the working memory paradigm with electrophysiological recordings. Participants were better able to remember the location for Black than White faces. During the encoding period, the P300 amplitude reflected differential attention to race, with larger amplitudes being associated with Black faces whose location was subsequently correctly remembered. In response to memory probe stimuli, the N170 component indicated enhanced processing of Black relative to White faces. Additionally, correct location responses elicited a larger P300 and LPC. Finally, greater probe LPC amplitudes were associated with better recollection of Black face location. Experiment 2 indicated that the effects from experiment 1 were not solely due to skin color differences since participants had better memory for Asian than White faces. However, both Asian and Caucasian participants had better memory for Asian than White faces, a finding inconsistent with the own-race bias.

By examining ERPs to encoding stimuli and subsequently sorting them by race and accuracy, we identified an electrophysiological signature of attention to the race of face stimuli. The assumption is that those faces whose location was correctly identified at probe were the ones most attended to at encoding. While previous research has found greater N200s for own-race than cross-race faces, indicating greater attention and individuation of own-race faces (Ito and Urland, 2003; Dickter and Bartholow, 2007; Kubota and Ito, 2007; Brebner et al., 2011; Lucas et al., 2011), our results did not indicate significant differences in N200s between White and Black probe trials. The weaker effect on N200 amplitudes during encoding is likely due to the simultaneous presentation of multiple faces. Because four faces were displayed at the same time during encoding, the precise timing of early processing as individuals were mentally moving through the stimuli could not be captured by ERPs, hence the weaker race effects on early visual processing components. Future research should use other methodology, such as eye tracking, to more precisely measure early attentional effects when viewing multiple racial stimuli simultaneously.

The main processing difference captured during encoding was reflected later in processing in the P300, a component associated with arousal and attention to motivationally significant events (Polich and Kok, 1995; Nieuwenhuis et al., 2005). Greater P300 amplitudes for Black correct and White incorrect trials suggest greater attentional allocation to Black faces overall, that likely resulted from increased motivation to attend to Black faces. These results are consistent with the proposal that attention is moderated by motivational factors (Brosch and Van Bavel, 2012; Correll et al., 2014) and that increased motivation to encode cross-race faces will result in better memory (Hugenberg et al., 2010). Thus, the greater P300 amplitudes at encoding for Black faces indicates increased motivation to encode Black faces and likely contributed to greater behavioral accuracy for Black than White face location. The greater motivation to attend to Black faces at encoding may be due to several factors, first participants were instructed to be as accurate as possible, thereby increasing motivation to remember cross-race faces, which naturally would be more difficult. Secondly, in line with previous research indicating greater P300s for emotionally valenced or more arousing stimuli (Eimer et al., 2003; Kubota and Ito, 2007), Black faces may have elicited greater emotional arousal relative to White faces, resulting in greater attention. Finally, it is possible that the intergroup context increased the saliency of race such that participants became aware that the task was targeted at some aspect of race. Perhaps with that framing in mind, egalitarian attitudes toward not appearing prejudiced may have motivated participants to encode and accurately remember Black faces. However, because egalitarian attitudes were not measured in the present study, future research will have to assess this possibility.

To investigate racial bias effects when a single stimulus was presented during retrieval, we examined the ERPs to probes. Experiment 1 indicated that the N170, an early visual processing component associated with perceptual processing of faces, was greater (i.e., more negative deflections) for Black than White faces. While the present N170 results are consistent with previous research (Herrmann et al., 2007; Stahl et al., 2008; Walker et al., 2008; Brebner et al., 2011; Wiese et al., 2014), it is important to note that previous work examining the effects of race on the N170 has produced mixed results. For example, some research has not found effects of race on the N170 (Caldara et al., 2003); while other research has found larger N170s to own-race than cross-race faces (Ito and Urland, 2005). It has been suggested that the mixed findings on the N170 may be due to differences between experimental tasks (Senholzi et al., 2015). In particular, when discrimination at the subordinate level is required (as in the present experiment), it is more likely that the N170 will be greater for cross-race than own-race faces because participants are motivated to attend to cross-race faces, which are more difficult to discriminate than own-race faces (Ito and Bartholow, 2009; Senholzi et al., 2015). Our findings are consistent with this proposal and suggest that the greater N170 amplitudes to Black faces reflect greater attentional resources allocated to Black than White faces. Moreover, motivation to attend to race may have increased since race was made salient through the intergroup context and error rates indicated that when individuals saw two Black and two White faces, racial identity was automatically encoded. Additionally, participants were instructed to be as accurate as possible, thereby increasing their motivation to encode the faces. Together with the accuracy results indicating better memory for Black than White faces, it is likely that greater N170 amplitudes reflect motivational factors during face processing.

In addition to the early probe effects that were sensitive to race, greater P300s were observed for correct relative to incorrect trials regardless of race. As stated previously, increased P300s are associated with greater attention to motivationally significant events (Brosch and Van Bavel, 2012; Correll et al., 2014). Thus, greater accuracy suggests increased information availability reflected through larger P300s. This accuracy effect also extended into the LPC, with greater amplitudes for correct relative to incorrect trials. The LPC is commonly examined in the memory literature and is associated with memory recognition processes (Sanquist et al., 1980; Paller et al., 1987; Van Petten and Senkfor, 1996). Correctly identified old stimuli elicit greater LPCs than new stimuli (Paller et al., 2003), reflecting greater recollection. Likewise, larger LPCs for correct than incorrect trials indexed successful retrieval. Importantly, larger LPCs for Black than White faces also indicated greater recollection and attentional capture for Black faces, which was also evident through better location accuracy for Black probes.

In experiment 2, both Asian and Caucasian participants had better location accuracy for Asian than White faces, a finding inconsistent with the own-race bias in memory. It is possible that these contradictory findings may be due to differences in the processes underlying working memory and long-term memory. In fact, a previous study examining working memory for Black and White faces did not find differences in memory for Black and White faces in White participants (Sessa et al., 2012). The incorporation of competition for attention between races in the present tasks suggests that our results may be better explained by visual search literature examining detection for own- and cross-race faces (Levin, 2000; Chiao et al., 2006; Sun et al., 2013). In fact, our results are consistent with work showing faster and more accurate detection of Black than White faces regardless of perceivers’ race (Chiao et al., 2006). Likewise, in the present experiments, it appears that participants were faster at detecting Black and Asian faces relative to White faces. Moreover, the greater attentional capture by Black and Asian faces relative to White faces was sustained throughout encoding. Thus, during intergroup contexts Black and Asian faces are detected more quickly and capture more attention relative to White faces.

In both experiments, error rate patterns were consistent with previous research indicating that perceivers tend to first automatically categorize individuals by race (Taylor et al., 1978; Dickter and Bartholow, 2007). The greater number of within-race than between-race errors indicate that participants encoded the race of the faces even though they were not instructed to attend to race. Moreover, participants made more within-race errors for White relative to Black or Asian faces, providing evidence of greater categorical processing for White faces. It has been proposed that greater categorical processing of faces results in poorer encoding and reduced memory (Levin, 2000; Hugenberg et al., 2010); therefore, greater categorization of White faces likely contributed to the reduced location accuracy for White faces. Altogether, our findings suggest that by simultaneously presenting competing racial stimuli (i.e., intergroup contexts), one can increase the salience of race. Moreover, the saliency of race increased attention to Black and Asian faces, as well as, categorization of White faces, resulting in worse encoding and recognition for White relative to Black and Asian faces.

One limitation of the present experiment is that participants were instructed to fixate on a central fixation cross due to the nature of the ERP experiment and the need to reduce artifacts due to eye movements. By asking participants to fixate on a cross, we may not have captured an individual’s natural patterns of visual attention. Although facial stimuli were placed at a visual angle that allowed all faces to be visible in a “window” of attention, forcing participants to fixate on a central location may not be completely ecologically valid. Thus, future studies should examine this issue using other technologies to measure visual attention (e.g., eye tracking). In the present experiments, there was no relationship between location accuracy and explicit racial attitudes, these findings are consistent with a previous meta-analysis indicating no relationship between explicit racial attitudes and own-race biases in memory (Meissner and Brigham, 2001). The present results did not indicate an own-race bias in working memory, nevertheless, these findings contribute to previous literature by showing no direct relationship between racial attitudes and working memory biases to cross-race faces. However, it has been proposed that racial attitudes may play a mediating role on memory for cross-race faces through other factors such as contact with other races (Meissner and Brigham, 2001). Thus, future research should examine whether racial attitudes mediate the relationship between contact with other races and working memory.

The present experiments sought to expand our knowledge of racially biased attention and memory during intergroup contexts by examining what occurs when there is competition for attention between multiple racial stimuli. Overall, our results indicate that when individuals are presented with own- and cross-race faces simultaneously, attention is captured by racial minority faces which results in better working memory for those faces. These findings add in important ways to our understanding of the basic cognitive processes that give rise to and support racially biased attention and memory during competition for attention. First, using paradigms that only present one race may not adequately capture racial biases in attention and memory, thereby limiting the scope of research on racially biased behavior during intergroup contexts. More importantly, presenting faces of multiple races simultaneously appears to have naturally drawn attention to racial minority groups, providing a unique approach for increasing attention to racial minorities without using negative or explicit motivation. Understanding how the saliency of race may affect other types of racially biased behavior at the personal and societal levels will be an important task to complete in future research.

## Data Availability Statement

All the data as well as the material is openly accessible at https://osf.io/h7sdf/?view_only=cb1bef04a40c4c23a02cb2317389f890.

## Ethics Statement

This study was carried out in accordance with the recommendations of the Human Subjects Research Training provided by the Office of Research Support and Compliance at The University of Texas at Austin with written informed consent from all subjects. All subjects gave written informed consent in accordance with the Declaration of Helsinki. The protocol was approved by the Institutional Review Board at The University of Texas at Austin.

## Author Contributions

GG performed the testing, data collection, and analysis, performed data interpretation under the supervision of DS, and drafted the manuscript. DS critically revised the manuscript. All authors contributed to the study concept and design, and approved the final version of the manuscript for submission.

## Conflict of Interest Statement

The authors declare that the research was conducted in the absence of any commercial or financial relationships that could be construed as a potential conflict of interest.

## Footnotes

1 Repeated measures ANOVAs were also conducted for only White participants (*n* = 25) to ensure that the effects that were found were still evident even after participants from other races (i.e., Asian and “Other”) were excluded, here we present results for the encoding P300 at the parietal location. Results for the parietal location did not indicate a significant main effect of race (*p* > 0.05) nor accuracy (*p* > 0.05) on P300 amplitudes. While the interaction between race and accuracy was not significant [*F*(1,24) = 2.66, *p* = 0.12, $\eta_{p}^{2}$ = 0.10] P300 amplitudes followed the same pattern as the overall sample (*n* = 35).

2 Results for White participants (*n* = 25) for the probe N170 followed the same pattern as the overall sample (*n* = 35). There was a significant main effect of hemisphere [*F*(1,24) = 5.64, *p* = 0.03, $\eta_{p}^{2}$ = 0.19] with more negative N170 amplitudes in the left than right hemisphere. Additionally, there was a significant main effect of race [*F*(1,24) = 11.28, *p* = 0.003, $\eta_{p}^{2}$ = 0.32] with more negative N170 amplitudes for Black than White probes. There were no other significant main effects (*p* > 0.05) nor interactions (*p* > 0.05) on N170 amplitudes for White participants.
